# Changes in Plasma Choline and the Betaine-to-Choline Ratio in Response to 6-Month Lifestyle Intervention Are Associated with the Changes of Lipid Profiles and Intestinal Microbiota: The ICAAN Study

**DOI:** 10.3390/nu13114006

**Published:** 2021-11-10

**Authors:** HanByul Jang, Hyunjung Lim, Kyung-Hee Park, SangIck Park, Hye-Ja Lee

**Affiliations:** 1 Division of Endocrine and Kidney Disease Research, Korea National Institute of Health, Cheongju 28159, Chungbuk, Korea; greatstar@korea.kr (H.J.); sooin0108@korea.kr (S.P.); 2 Department of Medical Nutrition, Kyung Hee University, Yongin 17104, Gyeonggi-do, Korea; hjlim@khu.ac.kr; 3 Department of Family Medicine, Hallym University Sacred Heart Hospital, Hallym University, Anyang 14068, Gyeonggi-do, Korea; beloved920@naver.com

**Keywords:** choline, betaine, metabolite, metabolic parameters, Firmicutes, gut microbiota

## Abstract

Trimethylamine *N*-oxide (TMAO) and its precursors, including choline, betaine, and L-carnitine, are gut microbiota-related metabolites associated with the risk of obesity. We aimed (1) to comprehensively examine whether the changes in plasma TMAO and its precursors induced by lifestyle intervention are associated with the improvements in plasma metabolic parameters; and (2) to identify the fecal microbiome profiles and nutrient intakes associated with these metabolites and metabolic index. Data from 40 participants (obese children and adolescents) having the plasma metabolites data related to the changes in BMI z-scores after 6-month lifestyle intervention were analyzed. In this study, we observed that choline and the betaine-to-choline ratio (B/C) showed different patterns depending on the changes in BMI z-scores by the response to lifestyle intervention. During the 6 months, an increase in choline and a decrease in B/C were observed in non-responders. We also found that changes in choline and B/C were associated with the improvements in plasma lipid levels. Individuals who showed reduced choline or increased B/C from the baseline to 6 months had a significant decrease in LDL-cholesterol over 6 months compared to those with increased choline or decreased B/C, respectively. In addition, the increase in choline or decrease in B/C was associated with the increase in plasma triglycerides. The distribution of gut microbiota belonging to the Firmicutes, such as Clostridia, Clostridiales, Peptostreptococcaceae, *Romboutsia*, and *Romboutsia timonensis* was altered to be lower during the 6 months both as choline decreased and B/C increased. Moreover, the decrease in choline and the increase in B/C were associated with reduced fat intake and increased fiber intake after the 6-month intervention. Finally, lower abundance of *Romboutsia* showed the association with lower LDL-cholesterol and higher intake of fiber. In summary, we demonstrated that reduced choline and increased B/C by lifestyle intervention were associated with the improvements of LDL-cholesterol and triglycerides, low-fat and high-fiber intakes, and low abundance of Firmicutes. These indicate that changes to circulating choline and B/C could predict individuals’ changes in metabolic compositions in response to the lifestyle intervention.

## 1. Introduction

The prevalence of obesity has steadily increased worldwide over the past few decades [1]. The high prevalence of obesity among children and adolescents is particularly concerning, as excessive adiposity during childhood is a major risk factor for adult obesity and associated with metabolic complications [2,3]. Fortunately, mounting evidence has demonstrated that successful weight loss prior to early adulthood reduces the future risk of obesity and metabolic disease [3,4]. These findings emphasize the importance of providing interventions early in life, while also suggesting the need for monitoring targets to predict adiposity reductions.

The gut microbiota has been recently identified as an important environmental factor associated with obesity and related metabolic disorders [5,6]. According to recent studies, obesity-related gut microbiota changes affect some circulating metabolites, such as trimethylamine *N*-oxide (TMAO) and its precursors, which play pivotal roles in regulating obesity and obesity-related diseases [7,8,9,10,11]. TMAO is an amine oxide mainly generated from the oxidation of trimethylamine (TMA), which intestinal bacteria produce from dietary choline, L-carnitine, betaine, and γ-butyrobetaine [9]. Previous studies have shown that changes in TMAO and its precursors, choline and L-carnitine, not only predict successful weight loss in overweight and obese adults but are also related to improvement in the insulin sensitivity [12]. Although recent research has demonstrated that changes in gut microbiota-related metabolites due to weight-loss programs are effective for treating obesity as well as improving body composition and glucose metabolism [11,12], few studies have analyzed the gut microbiota associated with such changes. Furthermore, few studies have examined whether the changes in metabolites induced by lifestyle interventions affect the changes in metabolic parameters and gut microbiota in children and adolescents.

In this study, we investigated whether circulating TMAO and its precursors, including choline, L-carnitine, and betaine, were affected by lifestyle interventions and whether changes in these metabolites were related to improvement in metabolic parameters among obese or severely obese children. In addition, we also examined the fecal microbiome profiles and nutrient intake associated with these metabolites.

## 2. Methods

### 2.1. Study Participants

The longitudinal Intervention for Childhood and Adolescent Obesity via Activity and Nutrition (ICAAN) study is a 2-year quasi-experimental intervention trial (cris.nih.go.kr, KCT0002718) to test interventions that can prevent excessive weight gain and improve several health indices in obese children and adolescents, which was conducted from August 2016 to May 2019. The study design and the effectiveness of the first 6 months of this program have previously been described [13]. In brief, 242 overweight, obese, and severely obese individuals were recruited into the ICAAN study and then assigned to three treatment groups as follows: usual care for obesity, including counseling on diet, physical activity and behavioral modification (usual care group); exercise intervention (exercise group); intensive nutritional and feedback intervention (nutritional group) group. Of the 242 participants, 40 subjects available for the measurement of TMAO and its precursors both at the baseline and at 6 months after intervention were included in this study. The BMI z-score of the participants tracked up to 6 months was used to divide the lifestyle intervention response group and the non-response group to find metabolic indicators showing differences between individuals who have or not the improving effect of obesity after intervention. The subjects whose BMI z-score was decreased after 6 months were allocated to the response group (*N* = 27), and subjects with sustained or increased BMI z-scores were allocated to the non-response group (*N* = 13). The study was approved by the Institutional Review Boards of Hallym University Sacred Heart Hospital (2016-I135) and the Korea Center for Disease Control and Prevention (2020-07-05-P-A). Written informed consent was obtained from all participants and their parents.

### 2.2. Measurements of BMI z-Score

Professionally trained personnel performed anthropometric examinations using a standardized protocol at baseline and 6-month intervention. Height and body weight were measured using a stadiometer (ds-103, DongSahn Jenix, Seoul, Korea). BMI was calculated as body weight in kilograms divided by height in meters squared and then converted to BMI z-score based on age- and sex-specific BMI values in the 2017 Korean growth chart for children and adolescents.

### 2.3. Dietary Assessments

Dietary intake of a total of 40 subjects at baseline and at 6-month intervention was estimated using a 3-day food record (2 weekdays and 1 weekend day). Parents or caregivers helped their children to complete the dietary records, and trained researchers checked whether the records contained sufficient information. Nutrient intakes were determined from food intakes using a computer-aided nutritional analysis program (CAN-Pro, Web version 5.0; The Korean Nutrition Society, Seoul, Korea, 2016)

### 2.4. Measurements of TMAO and Its Precursors

Fasting blood samples were obtained at baseline and 6 months. Metabolomic analysis was performed on 80 plasma samples (40 × 2 time points) using capillary electrophoresis time-of-flight mass spectrometry (CE-TOFMS) in two modes for cationic and anionic metabolites. Automatic integration software (MasterHands ver. 2.17.4.19 developed at Keio University) was used to obtain peak information, including *m*/*z*, migration time (MT), and peak area. We detected 194 putative metabolites (111 metabolites in cation mode and 83 metabolites in anion mode) based on the HMT (human metabolome technologies) standard library. Among 194 metabolites, we used gut microbiota-related metabolites, such as TMAO and its precursors (choline, betaine, l-carnitine) in the present study.

### 2.5. Microbiota Profiling through 16S rRNA Sequencing

Of the 40 total subjects, only DNA from 23 subjects who agreed to collection of feces at the baseline and 6 months were analyzed. Samples were collected by each subject at home. A fresh sample (~30 mL) was placed into a collection container with dry ice and stored at −70 °C in the laboratory prior to DNA extraction. DNA was extracted using the QIAamp DNA Stool Kit (Qiagen, Valencia, CA, USA) according to the manufacturer’s protocol.

The 16S rRNA gene fragments were amplified from the extracted DNA. The V3-V4 region of 16S RNA was analyzed and sequenced using the Illumina Miseq platform.

### 2.6. Analysis of Microbiota Sequencing Data

The analysis of sequencing data was conducted as previously described [14]. In brief, FASTAQ files were created for each sample using the index sequences, and then Illumina adapters were removed using the FASTP program. Error correction was performed on the region where the two reads overlap and paired-end datasets for each sample were merged using FLASH v1.2.11. For operational taxonomic unit (OTU) analysis, data affected by sequencing errors, such as merged sequences shorter than 400 bp or longer than 500 bp and raw reads with ambiguous sequences or chimera sequences, were removed. The number of OTUs was determined through clustering of the sequences from each sample using a 97% sequence identify cut-off in the CD-HIT-OUT program. Afterward, taxonomic assignment was performed using the Basic Local Alignment Search Tool (BLASTN v2.4.0, http://blast.ncbi.nlm.nih.gov/Blast.cgi (accessed on 5 March 2020)) and the corresponding reference database (National Center for Biotechnology Information, Bethesda, MD, USA). To increase the accuracy of the taxonomy assignment, no taxonomy was assigned in the case that the query coverage of the best matches to the database was less than 85% or the identity of the matched area was less than 85%.

### 2.7. Statistical Analysis

All statistical analyses were performed with the SAS software package (ver. 9.4; SAS Institute Inc., Cary, NC, USA), and values were expressed as mean ± standard deviation (SD) for continuous variables or as raw numbers and percentages for categorical variables. Variables with non-normal distributions based on the Shapiro–Wilk test were log-transformed prior to analysis. A paired *t*-test was used to compare the difference between pre-intervention (0 M) and post-intervention (6 months) within each group. Analysis of covariance (ANCOVA) was used to compare the between-group differences at 6 months post-intervention after adjustment for age, sex, BMI z-score, and values of the outcome traits at the baseline measurement. The differences in gut microbiota between 6-month change groups based on choline and B/C were determined using linear discriminant analysis effect size (LEfSe), and the cladogram generated from LEfSe analysis was used to identify the most differentially abundant taxa that are enriched in the microbiota of these groups. The correlations between changes in metabolic parameters, metabolites, nutrient intakes, and gut microbiota were assessed using Spearman’s partial correlation coefficients. A *p*-value of less than 0.05 was considered to indicate statistical significance.

## 3. Results

### 3.1. The Characteristics of Study Participants

In the present study, 40 obese subjects having plasma metabolites data before and after 6-month intervention were analyzed (Supplementary Table S1). The subjects whose BMI z-score was decreased after 6 months were allocated to the response group (*N* = 27), and subjects with sustained or increased BMI z-scores were allocated to the non-response group (*N* = 13). No significant differences were observed with age, sex, lipid and glycemic parameters (triglyceride, LDL-cholesterol, glucose, HOMA-IR), dietary intake (carbohydrate, protein, fat, and fiber) and type of intervention between groups at baseline (Supplementary Table S2). The baseline BMI z-scores of non-responders were slightly higher than those of responders. Except for change in BMI z-score, there were no differences between responders and non-responders in the changes of lipid and glycemic parameters and nutrient intakes before and after lifestyle intervention (Table 1).

### 3.2. Changes in TMAO and Its Precursors by the Responses to 6-Month Lifestyle Intervention

After intervention, we observed that the choline and the betaine-to-choline ratio (B/C) showed different patterns of change depending on the response to lifestyle intervention (Table 2). During the 6 months, an increase in choline and a decrease in B/C were observed only in non-responders, whereas no differences of plasma metabolites before and after intervention were observed in responders.

### 3.3. Association of Changes in Choline and B/C by Lifestyle Intervention with the Improvement in Metabolic Parameters

**Figure 1:**
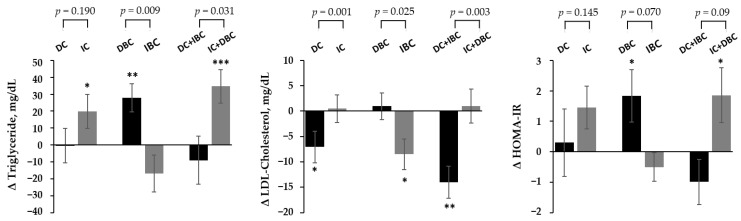
Changes in metabolic parameters by target metabolites group after lifestyle intervention. *p*-values were calculated using ANCOVA after adjustment for age, sex, baseline BMI z-score, values for the respective outcome traits at baseline to compare differences of between-groups at 6 months. Paired *t*-test was used to compare difference between pre-intervention (0 M) and post-intervention (6 months) within each group, and *p*-values less than 0.05 are marked with asterisks (* *p* < 0.05, ** *p* < 0.01, *** *p* < 0.001). DC, decreased choline group; IC, increased choline group; DBC, decreased betaine/choline group IBC, increased betaine/choline group.

Next, we analyzed whether the changes in plasma choline and B/C from the baseline to 6 months were associated with changes in plasma lipid and glycemic parameters. When subjects were divided into two groups according to whether choline and B/C increased or decreased after 6 months, we found that changes in choline and B/C are associated with plasma lipids (Figure 1 and Supplementary Table S3). The group of decreased choline or increased B/C was significantly associated with the reduction of LDL-cholesterol level at 6 months compared to those with increased choline or decreased B/C, respectively. In addition, when compared with decreased choline or increased B/C group, the group with increased choline or decreased B/C was associated with the increase in plasma triglycerides, respectively. The changes in LDL-cholesterol and triglycerides according to the changes in metabolites were more pronounced in the combined group of choline and B/C. The glucose and HOMA-IR increased in the B/C decrease group, but this was not significant when compared to the B/C increased group. There was no significant difference in glucose and HOMA-IR according to the combined group of choline and B/C.

The associations between choline or B/C and metabolic parameters at baseline were observed in only HOMA-IR (Supplementary Table S4).

### 3.4. Association of Changes in Choline and B/C by Lifestyle Intervention with Gut Microbiota

**Figure 2:**
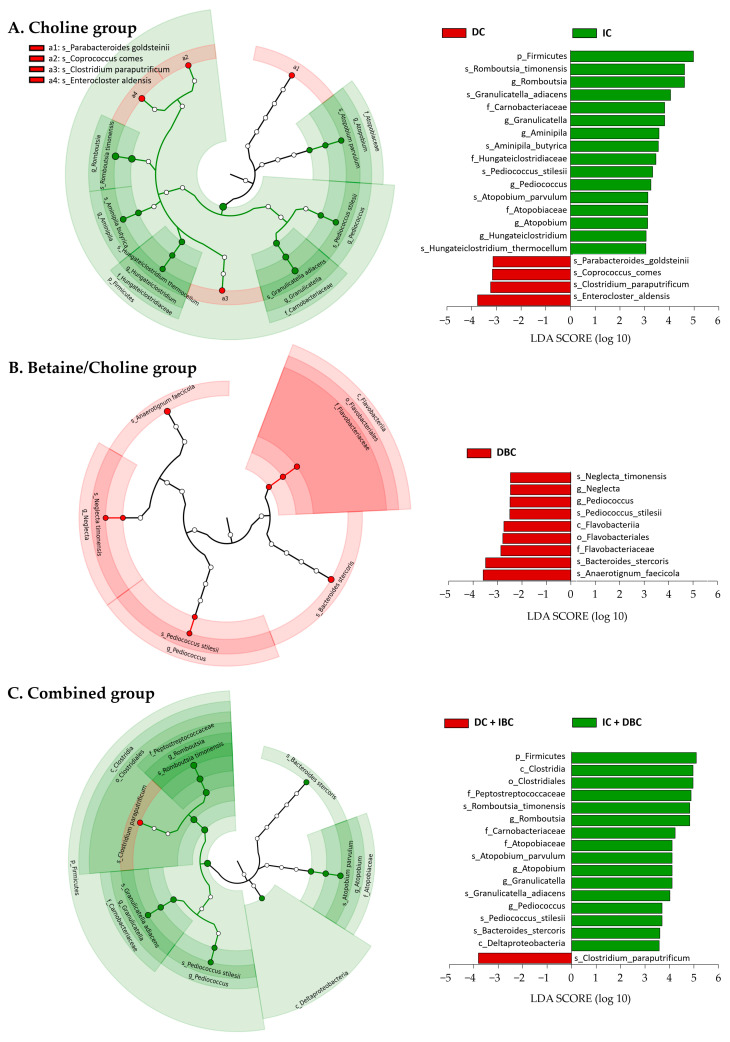
Intestinal microbiota taxa that discriminate between change groups (decrease vs. increase) in choline and B/C ratio after 6 months. Linear discriminant analysis with effect size (LEfSe) was performed using relative abundance data. Data shown are the log 10 linear discriminant analysis (LDA) scores following LEfSe analysis and the hierarchy of discriminating taxa visualized as cladograms for class comparisons between change groups of (**A**) choline, (**B**) betaine/choline, and (**C**) both choline and betaine/choline. DC, decreased choline group; IC, increased choline group; DBC, decreased betaine/choline group IBC, increased betaine/choline group.

Association analysis between changes in metabolites and gut microbiota was possible for only 23 subjects who donated feces among 40 subjects. The baseline clinical characteristics of these 23 subjects were no different from those of all 40 subjects (Supplementary Table S1). The histogram indicated that 32 phylotypes showed differences between the change groups associated with choline metabolism at 6 months (Figure 2). For example, Firmicutes was the dominant phylum in the increased choline group relative to the decreased choline group. Within this phylum, Carnobacteriaceae and Hungateiclostridiaceae at the family level and *Romboutsia*—*Romboutsia timonensis, Granulicatella—Granulicatella adiacens*, and *Aminipila—Aminipila butyrica* at the genus-species levels were identified as discriminating taxa for the increased choline group, while the species *Enterocloster aldensis* distinguished the decreased choline group (linear discriminant analysis, LDA, score ≥ 3.5). For the group with decreased B/C, *Anaerotignum faecicola* and *Bacteroides stercoris* were noted as discriminating taxa compared to the increased B/C group. In the group showing changes in both choline and B/C, the microbial features of the choline group were most prominent. Notable differences in the population of bacteria in the Firmicutes phylum were observed between these groups. Within the Firmicutes, Clostridia at the class level and its sub-level taxon Clostridiales–Peptostreptococcacea—*Romboutsia*—*Romboutsia timonensis* (order—family—genus—species), Carnobacteriaceae at the family level and its sub-level taxon *Granulicatella—Granulicatella adiacens,* and *Pediococcus* at the genus level and its species *Pediococcus stilesii* were more abundant in the group with both increased choline and decreased B/C. In addition, Atopobiaceae—*Atopobium*—*Atopobium parvulum* belonging to *Actinobacteria*, *Bacteroides stercoris* belonging to Bacteroidetes, and Deltaproteobacteria belonging to the Proteobacteria were identified as discriminating taxa for the group with both increased choline and decreased B/C, while *Clostridium paraputrificum* distinguished the group with decreased choline and increased B/C.

### 3.5. Association of Changes in Choline and B/C by Lifestyle Intervention with the Intake of Nutrients

**Figure 3:**
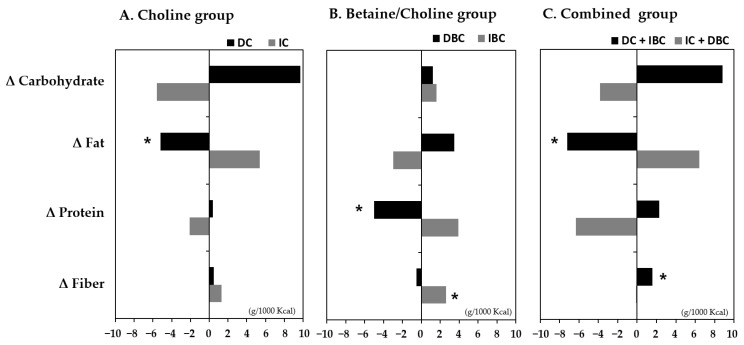
The association between dietary intake changes and target metabolites groups of (**A**) Choline, (**B**) Betaine/Choline, and (**C**) both choline and betaine/choline over a period of baseline to 6 months (Δ). In the paired *t*-test between pre-intervention (baseline) and post-intervention (6 months) values within each group, *p*-values less than 0.05 are marked with asterisks (*). DC, decreased choline group; IC, increased choline group; DBC, decreased betaine/choline group IBC, increased betaine/choline group.

We analyzed dietary intake to identify the nutrients associated with the changes in choline and B/C for 6 months (Figure 3). The decreased choline and B/C group was associated with the low intake of dietary fat and protein, respectively, and increased B/C was related to high fiber intake. When combining decreased choline and increased B/C, the significant association of fat and fiber intake with them was maintained.

### 3.6. Association of Microbiota Abundance with Metabolic Parameters and the Intake of Nutrients According to Lifestyle Intervention for 6 Months

**Figure 4:**
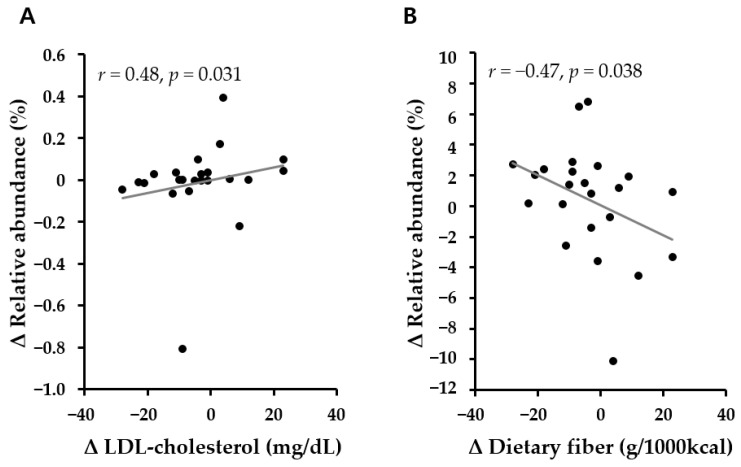
The correlation between changes in *Romboutsia* and (**A**) LDL-cholesterol and (**B**) dietary fiber over a period of baseline to 6 months (Δ). Spearman’s partial correlation analyses adjusted for age, sex, and baseline BMI z-score were performed.

To identify intestinal microbiota related to the changes of metabolic parameters and nutrients, Spearman’s partial correlation was tested after adjusted for age, sex, and baseline BMI z-score (Supplementary Figure S1). The abundance of *Romboutsia* only showed significant association with both the changes of LDL-cholesterol levels and fiber intake during the 6 months (Figure 4A,B). *Pediococcus* and *Neglecta timonensis* were associated with carbohydrate intake, and *Hungateiclostridium thermocellurr* showed the association with dietary fat intake (Supplementary Figure S1). The abundances of Flavobacteriia, Flavobacteriales, Flavobacteriaceae, and *Romboutsia* were negatively correlated with fiber intake, while *Clostridium paraputrificum* showed a positive correlation with higher intake of fiber during 6-month lifestyle intervention.

## 4. Discussion

In this study, we observed that choline increased and B/C decreased in the group with no response after 6 months of lifestyle intervention. Furthermore, we confirmed that the decrease in choline and increase in B/C in response to the reduction of the BMI z-score were associated with plasma lipid levels. Further, both decreased choline and increased B/C were associated with low fat and high fiber intake, and low abundance of Firmicutes.

Beneficial changes in choline and B/C associated with BMI z-score and lipid reductions were induced by lifestyle intervention in the present study. These results were generally consistent with those of previous studies, which reported that low levels of choline or high levels of betaine were associated with lower BMI, waist circumference, and body fat percentage [11,15]. In addition, a diet-induced weight loss study showed that decreases in choline, rather than L-carnitine and TMAO, were strongly associated with greater reductions in obesity indices over 6 months [11]. In our study, L-carnitine and TMAO showed no relationship with BMI z-score loss. Heianza et al. [11] hypothesized that because the production of TMAO from dietary quaternary amines (choline, l-carnitine, and betaine) is regulated by the gut microbiome and hepatic flavin-containing monooxygenases (FMOs), weight loss due to dietary changes would be more clearly reflected by precursors of TMAO than TMAO itself. Although both choline and l-carnitine are dietary precursors of TMAO, different pathways are involved in the formation of TMA from these two substances [12], which may explain our findings observed for choline and L-carnitine.

Prior observational studies [15,16,17] in the general population showed that low choline and high betaine levels were associated with favorable lipid and glycemic parameters. In the baseline result of our cross-sectional analysis, circulating lipid levels were not related to choline and B/C levels, and only HOMA-IR was positively related to choline (Supplementary Table S4). The differences from the previous results may have been due to the characteristics of the study population in that the subjects of our study were obese children and adolescents while those of previous studies were general adults [15,16,17]. However, when the change values of metabolic parameters over 6 months of intervention were analyzed, choline reduction showed the association with LDL-cholesterol reduction and B/C increase was associate with the decrease in triglycerides and LDL-cholesterol levels, suggesting that choline and B/C changes were closely related to changes in circulating lipid levels.

Although the mechanisms through which plasma levels of choline and betaine affect lipid metabolism remain unclear, their association can be explained in part through animal studies. Choline metabolism is divided into several pathways, which are related to the synthesis of betaine and TMA. Choline can be oxidized to betaine, which is a methyl donor for the epigenetic regulation of DNA [18]. Previous research indicated that betaine supplementation could reduce homocysteine levels, which are known to suppress lipolysis in adipocytes via transmethylation of homocysteine to methionine [19]. Moreover, high levels of betaine may be related to increased expression of PPAR-α and ACOX1/2, which are associated with fatty acid oxidation [20], or may reduce the capacity of fatty acid and triglyceride synthesis by decreasing the activities of sterol regulatory element-binding protein 1 (SREBP1), acetyl-CoA carboxylase (ACC), malic enzyme (ME), and fatty acid synthase (FAS) in adipose tissues [21,22].

Recent research has indicated that dietary choline and betaine may be metabolized by gut microbes to form TMA, which is further transformed into TMAO by hepatic FMOs [9]. A few studies of microbes related to choline metabolism have suggested that the phyla Firmicutes and Proteobacteria are involved in TMA production [23]. Although our study had a limitation on analysis of microbiota involved in TMA production due to lacking plasma TMA measurements, we found that phylum Firmicutes is dominant in the samples where choline increased during the 6-month period of intervention. Within the Firmicutes, Clostridia and its sub-level taxon Clostridiales—Peptostreptococcaceae—*Romboutsia*—*Romboutsia timonensis* (order—family—genus—species) were significantly enriched in the group showing both increased choline and decreased B/C, which is consistent with the finding of a previous animal study that plasma TMA has a positive correlation with Clostridiales [24]. *Romboutsia* has also been reported as an obesity-related phylotype, and it has been shown in obese mice that the abundance of *Romboutsia* is reduced with liraglutide treatment [25], a drug used to treat obesity and type 2 diabetes. Moreover, our finding that *Romboutsia* is positively related to LDL-cholesterol (Figure 4) supports a previous animal study that reduced levels of *Romboutsia* were associated with weight loss and decreased lipid levels [26]. Our microbiota analysis related to high- or low-TMAO producers was not available because no significant difference was found in plasma TMAO levels after intervention, although a previous study showed that the Firmicutes-to-Bacteroidetes ratio (F/B) was high in high-TMAO producers, who showed increases in urine TMAO of more than 20% in response to meals containing eggs and beef [27]. In addition, previous animal and human studies [28,29,30,31] reported that low-fat or high-fiber diet reduced TMA/TMAO metabolism and Firmicutes abundance. Similarly, we also observed that both a decrease in choline and increase in B/C in individuals showing a low abundance of gut microbiota belonging to the Firmicutes were associated with decreased fat and increased fiber intake in the present intervention study. Moreover, changes in dietary fiber intake were directly related to the change of *Romboutsia* belonging to Firmicutes. These findings suggest that dietary intervention can regulate the microbes involved in TMA production. We assume that reducing the proportion of bacteria that produce TMA from choline through dietary intervention and increasing the conversion from choline to betaine could help improve plasma lipids. Further well-designed clinical trials and animal studies are needed to validate this hypothesis, which may help to clarify the potential contributions of metabolites, gut microbiota, and diets to lipid profiles.

The present study has several strengths. To our knowledge, this is the first investigation measuring gut microbiota-related metabolites, gut microbiota, nutritional intake, and metabolic parameters at once. However, because we failed to collect feces from all subjects, we analyzed microbiota-related metabolites for a subset of subjects who agreed to collect fecal samples. In addition, in the analysis of metabolites using CE-TOFMS, TMA metabolites in plasma were not measured, making it impossible to identify the bacteria that produced TMA. Finally, we also tried to investigate whether the association of choline and B/C with the improvement of plasma lipids was modified by the type of intervention or low fat and high fiber diet, but the analysis was not possible due to the sample size. Therefore, further large-scale and well-designed intervention studies are needed to confirm our findings.

In summary, we identified that circulating choline and B/C were associated with lipid profiles during a 6-month lifestyle intervention. Individuals with both decreased choline and increased B/C were found to have low gut microbiota belonging to Firmicutes and also showed decreased fat and increased fiber in dietary intake. These results imply that changes in gut microbiota-related metabolites and dietary intake through lifestyle intervention help to improve body metabolic composition. However, further research is needed to confirm these findings.

## 5. Conclusions

In the present study, non-responders to intervention showed a pattern of increasing choline and decreasing B/C during the 6 months. We found that individuals who showed both reduced choline and increased B/C from the baseline measurement to 6 months had significant decreases in LDL cholesterol over 6 months compared to those with increased choline but decreased B/C. Furthermore, we found that individuals with both decreased choline and increased B/C had low gut microbiota belonging to Firmicutes, such as Clostridia, Clostridiales, Peptostreptococcaceae, *Romboutsia*, and *Romboutsia timonensis*. Moreover, we observed that the changes in plasma choline and B/C were related to dietary fat and fiber intake. Therefore, we suggest that changes made by lifestyle intervention in plasma metabolites and gut microbiota help to improve body metabolic parameters.

## Figures and Tables

**Table 1:** Changes in metabolic parameters in responses to the lifestyle intervention.

	Responder (*N* = 27)		Non-Responder (*N* = 13)		*p*-Value
	Diff ± SD	Paired *t*-Test	Diff ± SD	Paired *t*-Test	
Metabolic parameters (Δ Baseline—6 month)					
BMI z-score	–0.27 ± 0.28	<0.0001	0.22 ± 0.17	0.0007	<0.0001
Triglyceride ^§^, mg/dL	4.96 ± 37.3	0.3482	27.2 ± 61.3	0.0565	0.1074
HDL-C, mg/dL	0.04 ± 4.97	0.9694	−2.46 ± 7.09	0.2345	0.1955
LDL-C, mg/dL	−2.52 ± 12.7	0.3122	−2.00 ± 15.2	0.6444	0.5620
Glucose, mg/dL	1.22 ± 7.76	0.4207	5.69 ± 10.6	0.0762	0.4227
HOMA-IR ^§^	0.32 ± 3.22	0.8039	2.45 ± 4.62	0.0607	0.1038
Nutrients intake, g/1000 kcal/day (Δ Baseline—6 month)					
Carbohydrate	1.88 ± 20.1	0.7123	−0.08 ± 30.9	0.9953	0.1326
Protein	−1.06 ± 8.10	0.6095	−0.78 ± 61.3	0.8342	0.7425
Fat	−0.09 ± 7.80	0.9624	2.37 ± 7.09	0.6809	0.3709
Dietary fiber	1.07 ± 2.45	0.1007	0.48 ± 15.2	0.7803	0.2695

Abbreviations: Diff, Difference; BMI z-score, body mass index z-score; HDL-C, high-density lipoprotein cholesterol; LDL-C, low-density lipoprotein cholesterol; HOMA-IR, homeostasis model assessment of insulin resistance. ^§^ Variables were log-transformed prior to analysis; *p*-value was calculated by analysis of covariance (ANCOVA) to compare the between-group differences at 6 months post-intervention after adjustment for age, sex, and values of the outcome traits at baseline. Paired *t*-test was used to compare the difference between pre-intervention (0 M) and post-intervention (6 months) within each group.

**Table 2:** Changes in metabolites related to choline metabolism after lifestyle intervention.

	Responder (*N* = 27)		Non-Responder (*N* = 13)		*p*-Value
	Diff ± SD	Paired *t*-Test	Diff ± SD	Paired *t*-Test	
Choline metabolism related metabolites (Δ Baseline—6 month)					
Carnitine	−0.0127 ± 0.0488	0.189	−0.0177 ± 0.0293	0.051	0.493
Choline ^§^	0.0028 ± 0.0174	0.335	0.0102 ± 0.0153	0.035	0.119
TMAO ^§^	−0.0001 ± 0.0146	0.840	0.0001 ± 0.0055	0.850	0.233
Betaine	−0.0062 ± 0.0479	0.509	−0.0027 ± 0.0442	0.830	0.564
Betaine/Choline ^§^	−1.4030 ± 5.6760	0.192	−2.3440 ± 3.1800	0.024	0.660
TMAO/Choline	0.0270 ± 1.3670	0.919	0.0809 ± 0.4270	0.508	0.283

Abbreviations: Diff, Difference; TMAO, trimethylamine *N*-oxide. Data are expressed as mean differences ± SD (×0.1); ^§^ Variables were log-transformed prior to analysis; *p*-value was calculated by analysis of covariance (ANCOVA) to compare the between-group differences at 6 months post-intervention after adjustment for age, sex, baseline BMI z-score and values of the outcome traits at baseline. Paired *t*-test was used to compare the difference between pre-intervention (baseline) and post-intervention (6 months) within each group.

## Data Availability

Sharing data publicly is restricted by the Ethics Committee of the Korea Center for Disease Con-trol and Prevention (KCDC) because there is no third-party agreement on personal information. Data requests can be submitted to the corresponding author (H.L., hyejalee@korea.kr).

## Supplementary Materials

The following are available online at https://www.mdpi.com/article/10.3390/nu13114006/s1, Figure S1: The correlation between changes in microbiota and metabolic parameters and dietary intake over period of baseline to 6 months, Table S1: Results of one-sample *t*-test for participants, Table S2: Baseline characteristics by the response to lifestyle intervention, Table S3: Changes in metabolic parameters according to target metabolites group related to 6 month lifestyle intervention, Table S4: Associations of plasma choline or betaine/choline with metabolic parameters at baseline.
