# Supplementary material for: Changes in Plasma Choline and the Betaine-to-Choline Ratio in Response to 6-Month Lifestyle Intervention Are Associated with the Changes of Lipid Profiles and Intestinal Microbiota: The ICAAN Study
Source: Nutrients. 2021 Nov 10;13(11):4006. doi: 10.3390/nu13114006 (PMC8625635; doi:10.3390/nu13114006)
Supplement: Supplementary file 1 [file nutrients-13-04006-s001.zip › nutrients-1429068-supplementary.pdf]

Supplementary materials

Supplemantary Table S1. Results of one-sample *t*-test for participants

	Metabolites set n = 40	Microbiota set n = 23	*P*
	Mean ± SD	Mean ± SD	
Age	11.1 ± 2.2	10.8 ± 2.3	0.5938
Boy, n(%)	21 (52.5)	12(52.2)	
BMI z-score	3.00 ± 1.00	3.07 ± 0.76	0.6520
Whole body fat, %	41.6 ± 4.4	41.8 ± 4.3	0.8395
Trunk fat, %	44.6 ± 5.1	44.8 ± 4.6	0.8261
Triglycerides	98.2 ± 45.8	98.1 ± 41.8	0.9914
HDL-cholesterol	51.3 ± 12.1	50.2 ± 10.8	0.6431
LDL-cholesterol	114.7 ± 20.5	118.8 ± 22.2	0.3799
Glucose	87.9 ± 7.1	86.4 ± 7.0	0.3212
HOMA-IR	4.0 ± 2.2	4.1 ± 1.9	0.8040

Supplementary Table S2. Baseline characteristics by the response to lifestyle intervention

	All (N=40)	Responder (N=27)	Non-responder (N=13)	*P*-values
Age	11.1 ± 2.2	10.85 ± 1.99	11.61 ± 2.71	0.3244
Boy, n (%)	21 (52.5)	14 (51.9)	7 (53.9)	0.9058
BMI z-score	3.00 ± 1.00	2.75 ± 0.76	3.52 ± 1.24	0.0380
Whole body fat, %	41.6 ± 4.4	41.2 ± 4.68	42.3 ± 3.88	0.4296
Trunk fat, %	44.6 ± 5.1	44.3 ± 5.40	45.3 ± 4.55	0.5811
Lipid and glycemic parameters				
Triglyceride §, mg/dL	98.2 ± 45.8	96.3 ± 45.2	102.3 ± 48.5	0.7997
HDL-cholesterol, mg/dL	51.3 ± 12.1	51.6 ± 13.9	50.6 ± 7.35	0.9524
LDL-cholesterol, mg/dL	114.7 ± 20.5	112.8 ± 23.5	118.5 ± 12.3	0.4881
Fasting glucose, mg/dL	87.9 ± 7.1	89.1 ± 7.08	85.4 ± 6.64	0.1040
HOMA-IR §	4.0 ± 2.2	3.94 ± 2.37	4.17 ± 1.97	0.7023
Nutrients intake, g/1,000 Kcal/day				
Carbohydrate	142.7 ± 22.1	144.2 ± 21.9	138.3 ± 24.2	0.6232
Protein	30.2 ± 7.15	29.8 ± 7.46	31.1 ± 6.77	0.8185
Fat	38.1 ± 8.78	37.9 ± 8.26	38.6 ± 11.0	0.7957
Dietary fiber	8.30 ± 2.20	8.48 ± 2.19	7.81 ± 2.37	0.5743
Intervention(Uc/ Ex/ Nu), n (%)	10/ 15/ 15 (25.0/ 37.5/ 37.5)	5/ 13/ 9 (18.5/ 48.2/ 33.3)	5/ 2/ 6 (38.5/ 15.4/ 46.1)	0.1169

Abbreviations: BMI, body mass index; Uc, usual care group; Ex, exercise group; Nu, Nutritional group

§ Variables were log-transformed prior to analysis

Data are expressed as the mean ± SD or n (%); *P* values were calculated through generalized linear regression analysis with age and sex for continuous parametric variables and Chi-square test for categorical variables.

Supplementary Table S3. Changes in metabolic parameters according to target metabolites group related to 6 month lifestyle intervention

	Difference ± SD	Paired t-test	Difference ± SD	Paired t-test	ANCOVA
Choline group	DC (N=15)		IC (N=25)		
BMI z-score	-0.14 ± 0.37	0.1718	-0.09 ± 0.32	0.1582	0.5963
Triglyceride §, mg/dL	-0.40 ± 39.79	0.766	19.8 ± 49.9	**0.0282**	0.1899
HDL-cholesterol, mg/dL	-1.93 ± 5.22	0.1731	-0.08 ± 6.08	0.9481	0.3786
LDL-cholesterol, mg/dL	-7.07 ± 12.16	**0.0409**	0.48 ± 13.5	0.8604	**0.0012**
Fasting glucose, mg/dL	4.67 ± 10.84	0.1177	1.48 ± 7.48	0.3326	0.3283
HOMA-IR §	0.30 ± 4.28	0.4604	1.45 ± 3.51	**0.0283**	0.1445
Betaine/Choline group	DBC (N=26)		IBC (N=14)		
BMI z-score	-0.12 ± 0.38	0.1202	-0.09 ± 0.25	0.1864	0.5302
Triglyceride §, mg/dL	27.9 ± 42.7	**0.0013**	-16.8 ± 40.9	0.3571	**0.0091**
HDL-cholesterol, mg/dL	-1.58 ± 5.25	0.138	0.71 ± 6.59	0.6917	0.2628
LDL-cholesterol, mg/dL	0.96 ± 13.5	0.719	-8.5 ± 11.19	**0.0139**	**0.0254**
Fasting glucose, mg/dL	4.15 ± 8.91	**0.0254**	-0.07 ± 8.50	0.9754	0.4752
HOMA-IR §	1.83 ± 4.37	**0.0404**	-0.50 ± 1.74	0.3391	0.0701
Combined group	DC + IBC (N=8)		IC + DBC (N=19)		
BMI z-score	-0.13 ± 0.31	0.2801	-0.11 ± 0.36	0.2045	0.9903
Triglyceride §, mg/dL	-9.00 ± 40.4	0.9382	34.6 ± 42.8	**0.0005**	**0.0313**
HDL-cholesterol, mg/dL	-1.63 ± 6.76	0.5184	-1.32 ± 5.89	0.3430	0.9118
LDL-cholesterol, mg/dL	-14.0 ± 8.83	**0.0029**	1.00 ± 14.6	0.7687	**0.0026**
Fasting glucose, mg/dL	2.13 ± 9.88	0.5621	2.89 ± 7.53	0.1109	0.7462
HOMA-IR §	-0.99 ± 2.09	0.2068	1.85 ± 3.94	**0.0213**	0.0941

§ Variables were log-transformed prior to analysis

Analysis of covariance (ANCOVA) was used to compare the between-group differences at the time point of 6 months post-intervention after adjustment for age, sex, BMI z-score and values of the outcome traits at the baseline measurement. Paired t-test was used to compare the between pre-intervention (0M) and post-intervention (6 Months) difference within each group

DC, Decreased Choline group; IC, Increased Choline group; DBC, Decreased Betaine/Choline group IBC, Increased Betaine/Choline group

Supplementary Table S4. Associations of plasma choline or betaine/choline with metabolic parameters at baseline

	Baseline Choline			Baseline Betaine/Choline		
	ß	SE	*P*	ß	SE	*P*
Baseline						
Triglyceride	1.039	0.552	0.068	-0.500	0.355	0.168
HDL-cholesterol	0.071	0.283	0.803	-0.153	0.176	0.393
LDL-cholesterol	0.292	0.225	0.202	-0.201	0.141	0.163
Fasting glucose	-0.116	0.093	0.219	0.002	0.060	0.972
HOMA-IR	1.266	0.530	**0.022**	-0.152	0.359	0.675

ß (SE) for each outcome per 1 SC increment of log-transformed choline and betaine/choline after controlling for age sex, BMI z-score and values of metabolic parameters were log-transformed before analysis.

Δ Relative abundance (%)	Δ Metabolic parameters (mg/dL)					Δ Dietary intake, g/1,000 Kcal/day			
	**TG**	**HDL**	**LDL**	**HOMA-IR**	**Glucose**	**Carbohydrate**	**Fat**	**Protein**	**Fiber**
p_Firmicutes	0.04	0.06	-0.01	0.37	0.23	-0.30	0.01	0.08	-0.07
p_Bacteroidetes	0.04	-0.21	-0.13	-0.13	0.00	0.32	-0.14	-0.13	0.08
c_Clostridia	-0.04	-0.17	-0.24	0.12	-0.04	-0.31	-0.16	0.07	-0.23
c_Deltaproteobacteria	0.12	0.21	0.29	0.35	0.22	-0.02	-0.01	-0.27	-0.22
c_Flavobacteriia	0.18	-0.17	0.25	-0.32	-0.23	-0.12	-0.15	-0.35	**-0.47 ***
o_Clostridiales	-0.04	-0.17	-0.24	0.12	-0.04	-0.31	-0.16	0.07	-0.23
o_Flavobacteriales	0.18	-0.17	0.25	-0.32	-0.23	-0.12	-0.15	-0.35	**-0.47 ***
f_Atopobiaceae	0.15	-0.22	0.11	0.37	0.20	-0.32	0.44	0.03	0.03
f_Carnobacteriaceae	0.16	0.20	0.40	0.22	0.22	0.07	0.29	0.17	-0.12
f_Flavobacteriaceae	0.18	-0.17	0.25	-0.32	-0.23	-0.12	-0.15	-0.35	**-0.47 ***
f_Hungateiclostridiaceae	0.22	0.17	0.08	0.05	-0.22	0.11	0.20	-0.12	-0.18
f_Peptostreptococcaceae	-0.10	-0.01	0.41	-0.42	-0.32	-0.32	0.04	-0.11	-0.44
g_Aminipila	0.31	0.16	0.01	0.33	0.11	0.17	-0.19	-0.32	-0.13
g_Atopobium	0.15	-0.22	0.11	0.37	0.20	-0.32	0.44	0.03	0.03
g_Granulicatella	0.16	0.20	0.40	0.22	0.22	0.07	0.29	0.17	-0.12
g_Hungateiclostridium	0.27	0.12	0.07	0.02	-0.22	0.10	0.23	-0.14	-0.16
g_Neglecta	0.11	-0.13	-0.04	0.10	0.33	0.46	-0.31	-0.08	0.02
g_Pediococcus	-0.10	-0.10	0.35	-0.38	-0.32	**-0.39 ***	0.24	-0.17	-0.22
g_Romboutsia	-0.07	-0.03	**0.48 ***	-0.40	-0.24	-0.22	0.00	-0.21	**-0.47 ***
s_Aminipila butyrica	0.31	0.16	0.01	0.33	0.11	0.17	-0.19	-0.32	-0.13
s_Anaerotignum faecicola	0.16	-0.04	0.18	-0.18	-0.11	0.04	0.20	-0.14	-0.26
s_Atopobium parvulum	0.15	-0.22	0.11	0.37	0.20	-0.32	0.44	0.03	0.03
s_Bacteroides stercoris	0.33	-0.35	0.21	-0.33	-0.12	-0.19	0.14	-0.26	-0.28
s_Clostridium paraputrificum	-0.39	0.28	-0.38	-0.08	-0.17	-0.10	-0.10	0.39	**0.51 ***
s_Coprococcus comes	0.08	0.18	-0.10	0.22	0.25	0.05	0.33	0.17	0.14
s_Enterocloster aldensis	-0.15	-0.13	0.10	-0.03	0.21	0.17	-0.04	-0.03	-0.13
s_Granulicatella adiacens	0.18	0.19	0.41	0.18	0.16	0.10	0.27	0.17	-0.15
s_Hungateiclostridium thermocellur	0.04	0.29	0.11	-0.08	-0.33	-0.03	**0.47 ***	0.03	-0.01
s_Neglecta timonensis	0.11	-0.13	-0.04	0.10	0.33	**0.46 ***	-0.31	-0.08	0.02
s_Parabacteroides goldsteinii	0.08	-0.43	0.13	-0.12	0.09	0.22	0.16	-0.06	0.15
s_Pediococcus stilesii	-0.10	-0.10	0.35	-0.38	-0.32	-0.39	0.24	-0.17	-0.22
s_Romboutsia timonensis	-0.07	-0.03	**0.48 ***	-0.40	-0.24	-0.22	0.00	-0.21	**-0.47 ***

Supplementary Figure S1. The correlation between changes in microbiota and metabolic parameters and dietary intake over period of baseline to 6 months (Δ). Spearman's partial correlation analyses were adjusted for age, sex, and baseline BMI z-score, and p-values less than 0.05 are marked with asterisks (*).
